# Successful Management of Meningitis and Sepsis Due to Multidrug-Resistant Elizabethkingia meningoseptica in an Infant

**DOI:** 10.7759/cureus.101368

**Published:** 2026-01-12

**Authors:** Minat Allah Alhusami, Shahad Alshamsi, Amina Mujahid, Arun Jayaraman

**Affiliations:** 1 Medicine, Mohammed Bin Rashid University, Dubai, ARE; 2 Pediatrics, Mediclinic Middle East, Dubai, ARE

**Keywords:** elizabethkingia meningoseptica, multidrug resistance, neonatal meningitis, neonatal sepsis, neonatology, pediatric infectious disease, pediatric meningitis, pediatric neurology, pediatrics, pediatric sepsis

## Abstract

We report a case of a two-month-old male infant, born prematurely, who initially presented with fever, irritability, and elevated inflammatory markers. He was then diagnosed with *Elizabethkingia meningoseptica *meningitis and sepsis. Treatment included intravenous levofloxacin, ampicillin, and cefotaxime. Despite the bacterium’s multidrug resistance and high reported morbidity and mortality rates, the patient made a full recovery with no complications at discharge or follow-up. This case demonstrates the challenges in treating *E. meningoseptica *infections and highlights the potential clinical efficacy of antibiotic regimens that may not align with standard susceptibility testing.

## Introduction

*Elizabethkingia meningoseptica* (formerly *Chryseobacterium meningosepticum* and initially described as *Flavobacterium meningosepticum*) is a non-motile, non-fermentative, oxidase-positive Gram-negative bacillus. This opportunistic pathogen is widely distributed in nature, colonizing soil, water supplies, and medical devices. In hospital environments, it can persist in sink basins, water taps, and medical equipment, leading to nosocomial infections, particularly in neonates and immunocompromised individuals [1]. In neonates, *E. meningoseptica* is primarily associated with outbreaks of fatal meningitis, particularly in premature infants. However, it can also cause pneumonia, endocarditis, and bacteremia, especially in immunocompromised elderly patients.

Many *E. meningoseptica* strains produce two types of β-lactamases: class A extended-spectrum β-lactamases and class B metallo-β-lactamases (MBLs), conferring resistance to most β-lactams, including carbapenems, as well as aminoglycosides [2]. However, the bacterium often exhibits susceptibility to antibiotics typically used against Gram-positive bacteria, such as rifampicin, ciprofloxacin, and trimethoprim-sulfamethoxazole (TMP-SMX), with variable responses to vancomycin [1,3].

A retrospective chart review study conducted by Alsayed et al. contains the only cases reported in the literature in the United Arab Emirates (UAE) with this pathogen. Twenty-three cases, 10 of which were pediatric, were identified between 2017 and 2023, predominantly affecting immunocompromised neonates and the elderly. Notably, 91% exhibited multidrug resistance, with sensitivity primarily to quinolones, piperacillin-tazobactam, and TMP-SMX. Their findings agreed with existing literature identifying that formerly premature pediatric patients were at particular risk due to their immunocompromised status and exposure to hospital equipment, where this pathogen commonly resides. Five of the 10 children required admission to the intensive care unit, and mortality occurred in two [4].

We report a case of *E. meningoseptica* meningitis and sepsis in a premature infant, who demonstrated an excellent outcome following treatment with ampicillin, cefotaxime, and levofloxacin. To our knowledge, this is the first reported case of *E. meningoseptica* infection at this particular institution. While Alsayed et al. recently described a series of *E. meningoseptica* infections in both adult and pediatric populations across major hospitals in the UAE, our case was encountered at a separate facility not included in their study and therefore represents an independent occurrence [4].

This article was previously presented as a conference poster at the Third Mediclinic Pediatric Subspecialties Conference on April 11, 2025.

## Case presentation

A two-month-old male infant presented to a tertiary care hospital’s pediatric outpatient department with a one-day history of fever and irritability. There were no respiratory or gastrointestinal symptoms. He had been born prematurely at 33 weeks via cesarean section due to placenta previa and had a birth weight of 2 kg. His postnatal course included an eight-day stay at the neonatal intensive care unit (NICU) for ventilation, but he had been healthy and thriving on exclusive breastfeeding prior to this presentation.

On admission, he was started on intravenous (IV) cefotaxime and ampicillin, both at 300 mg/kg/day in three divided doses for the empiric management of suspected meningitis, along with antipyresis using oral paracetamol. Hydration was maintained with oral breastfeeding, supplemented with IV 0.9% sodium chloride at 150 mL/day. Meanwhile, a full diagnostic workup was initiated. Blood and cerebrospinal fluid (CSF) cultures revealed *E. meningoseptica*, with initial laboratory findings showing an elevated C-reactive protein (CRP) of 74.4 mg/L (normal: 0-5 mg/L), procalcitonin (PCT) of 24.8 ng/mL (normal: <0.05 ng/mL), and a white blood cell (WBC) count of 21.77 × 10³/L (normal: 5-18 × 10³/L) (Table 1). The CSF appeared turbid, with an elevated WBC count of 5,866.1 cells/mm³ (normal: 0-20 cells/mm³), protein level of 3,360 mg/L (normal: 150-450 mg/L), and glucose level of 0.21 mmol/L (normal: 3.33-4.44 mmol/L) (Table 2).

**Table 1:** Peripheral Blood Investigations During Hospitalization Values show initial elevated inflammatory markers and leukocytosis with a neutrophilic predominance, consistent with systemic bacterial infection, that improved by discharge. CRP: C-reactive protein; PCT: procalcitonin; WBC: white blood cell

Parameter	Reference range	On admission	Day 3 of admission	At discharge
CRP (mg/L)	0–5	74.4	147	<0.6
PCT (ng/mL)	<0.05	24.8	4.48	0.08
WBC (×10³/μL)	5–18	21.77	11.71	13.6
Neutrophils (%)	9.2–45.3	48.94	38.93	23.48
Lymphocytes (%)	34.7-81.6	36.51	52.41	59.01
Monocytes (%)	4.6-12.1	13.56	8.07	8.17

**Table 2:** Cerebrospinal Fluid Investigations During Hospitalization Findings indicate initial markedly low glucose, elevated protein, and elevated WBCs with neutrophilic predominance, consistent with bacterial meningitis, that improved by discharge. Monocytes were not detected in the differential count on admission. RBC: red blood cell; WBC: white blood cell

Parameter	Reference range	On admission	At discharge
Glucose (mmol/L)	3.33–4.44	0.21	1.85
Protein (mg/L)	150–450	3,360	1,715
RBC (cells/mm³)	0–2	648.4	1,002.1
WBC (cells/mm³)	0–20	5,866.1	125.9
Neutrophils (%)	0–6	65	10
Lymphocytes (%)	40-80	35	80
Mononuclear cells (%)	15-45	0	10

By the third day of hospitalization, the patient was clinically improving and hemodynamically stable, although he remained intermittently febrile and irritable with a tense, bulging anterior fontanelle. Supportive care included IV hydration and oral paracetamol, as oxygenation, perfusion, and oral feeding remained adequate, and the child remained seizure-free. Repeat laboratory investigations revealed a decrease in WBC and PCT, although CRP remained elevated. Antibiotic sensitivity testing of the blood culture showed resistance to cephalosporins (ceftazidime, cefotaxime, and cefepime) but susceptibility to doxycycline, minocycline, levofloxacin, and ciprofloxacin (Table 3).

**Table 3:** Blood Culture and Sensitivity Report The blood culture grew *Elizabethkingia meningoseptica.* The isolate was sensitive to fluoroquinolones and tetracyclines, but resistant to third- and fourth-generation cephalosporins.

Ciprofloxacin	Doxycycline	Levofloxacin	Minocycline	Cefepime, cefotaxime, ceftazidime
Sensitive	Sensitive	Sensitive	Sensitive	Resistant

**Figure 1:**
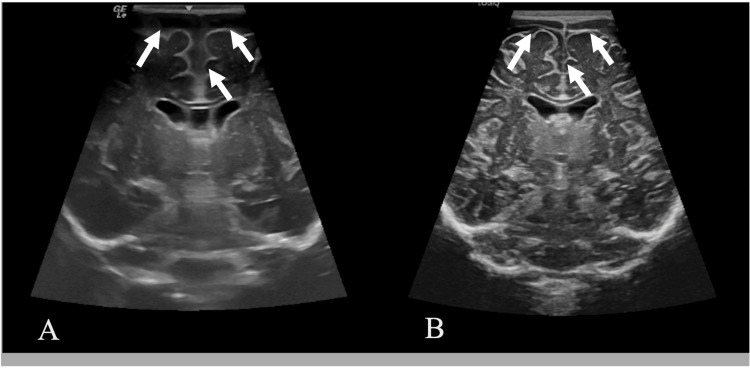
Cranial Ultrasounds at Admission and Two Months After Initiation of Treatment At admission (A), bifrontal and anterior hemispheric extra-axial cerebrospinal fluid spaces were prominent (arrows). On follow-up two months after treatment initiation (B), there was a marked reduction in the prominence of these spaces (arrows), consistent with therapeutic response.

However, when CSF culture results became available, the organism was found to be resistant to doxycycline, TMP-SMX, and cefotaxime but sensitive to levofloxacin (minimum inhibitory concentration (MIC): 0.25 μg/mL) (Table 4). Brain magnetic resonance imaging (MRI) revealed diffuse meningeal enhancement with bifrontal subdural collections and septations, with no evidence of infarction. Cranial ultrasound showed prominent bifrontal and anterior hemispheric CSF spaces (Figure 1A). Given the severity of central nervous system infection, radiological evidence of subdural collections, and discordant susceptibility profiles between blood and CSF isolates, combination antimicrobial therapy was deliberately continued. Additionally, IV levofloxacin was initiated at 20 mg/kg/day in two divided doses, and high-dose cefotaxime and ampicillin were maintained alongside levofloxacin to ensure sustained central nervous system antimicrobial coverage during the acute phase of illness, following discussion with pediatric infectious disease specialists. Supportive care was limited to supplementary IV fluids; no ongoing antipyretic therapy was required after initial defervescence. This strategy aimed to balance antimicrobial stewardship with clinical safety in a critically ill infant with complicated meningitis. All antibiotics were continued for a total of four weeks. IV hydration was continued at the same rate, and oral paracetamol was stopped as the patient had defervesced. Over the first two weeks of admission, he remained afebrile, demonstrated normal weight gain, and had no signs of neurological dysfunction. Serial cranial ultrasounds, electroencephalogram (EEG), and hearing assessments were all normal. At discharge, the patient had been afebrile for three weeks, showed normal head growth, and had improved blood and CSF investigations, three negative blood cultures, and one negative CSF culture (Tables 1, 2). A follow-up cranial ultrasound also demonstrated improvement of previously abnormal findings (Figure 1B). At one-year follow-up, the child was developmentally normal and remained stable and active, with no neurological deficits, hydrocephalus, or complications.

**Table 4:** Cerebrospinal Fluid Culture and Sensitivity Report The cerebrospinal fluid culture showed a different sensitivity profile compared to the blood isolate, remaining sensitive to fluoroquinolones and minocycline but resistant to doxycycline and cephalosporins, suggesting variable resistance expression between compartments.

Ciprofloxacin	Doxycycline	Levofloxacin	Minocycline	Cefotaxime
Sensitive	Resistant	Sensitive	Sensitive	Resistant

## Discussion

*E. meningoseptica* is a rare but serious cause of nosocomial infections in neonates and infants. Between 1944 and 2017, only 283 pediatric cases were reported, with just 6.7% occurring in infants aged 1-12 months [5]. In this population, *E. meningoseptica* primarily causes meningitis, with a mortality rate exceeding 50%. Survivors frequently develop severe complications such as hydrocephalus, brain abscesses, and developmental delays. Other infections associated with this bacterium include pneumonia and bacteremia [6].

The route of transmission for *E. meningoseptica* in the context of meningitis and sepsis is primarily nosocomial, most often via direct or indirect contact with contaminated environmental surfaces, hospital water sources, and medical equipment, including incubators, ventilators, intubation tubing, and humidifiers [6]. Our patient had an eight-day NICU stay for ventilation, which may have resulted in exposure through contact with medical equipment; however, no environmental investigation was pursued, as this hospitalization occurred at a different facility. The infant had no prior hospitalizations beyond this NICU stay and no known immunodeficiencies. 

Patient factors can also substantially increase susceptibility to this pathogen. Prematurity and low birth weight are well-recognized risk factors for *E. meningoseptica* infections, as preterm infants receive fewer maternal antibodies, leaving them more vulnerable to opportunistic pathogens [7]. Our patient was born prematurely at 33 weeks with a birth weight of 2 kg, further increasing susceptibility.

The treatment of *E. meningoseptica* is particularly challenging due to its extensive antimicrobial resistance. Standard empirical regimens for neonatal sepsis and meningitis, including β-lactams, aminoglycosides, and vancomycin, are largely ineffective against this pathogen [1,3,8,9]. This bacterium produces two β-lactamases, class A extended-spectrum β-lactamases and class B MBLs, contributing to its resistance to nearly all β-lactams, including cephalosporins and carbapenems. Susceptibility testing is further complicated by the lack of established MIC breakpoints, necessitating broth microdilution for accurate assessment [9].

In our case, CSF analysis revealed that the isolate was resistant to TMP-SMX, doxycycline, and third- and fourth-generation cephalosporins (ceftazidime, cefotaxime, and cefepime) but was sensitive to levofloxacin, minocycline, and ciprofloxacin. It was sensitive to doxycycline in serum but resistant in CSF, which may be attributed to methodological limitations in antimicrobial susceptibility testing, including the absence of standardized interpretive breakpoints for *E. meningoseptica* [10]. Additionally, multiple in vitro susceptibility studies confirm that *Elizabethkingia* species often have variable results across isolates, including for tetracyclines and other classes [11].

Interestingly, Alsayed et al. observed a notable difference in TMP-SMX susceptibility between different age groups infected with this pathogen in the UAE. While *E. meningoseptica* isolates in adult patients often retained susceptibility to TMP-SMX, none of the pediatric isolates demonstrated such susceptibility [9]. This aligns with our case, as the strain isolated from our pediatric patient was also resistant to TMP-SMX. 

Despite documented resistance, the patient received cefotaxime in combination with ampicillin and levofloxacin. Notably, his clinical improvement, along with a significant reduction in PCT and WBC count, suggests a potential discrepancy between in vitro resistance patterns and in vivo clinical response in this microbe. This raises important questions about whether in vitro susceptibility testing accurately predicts treatment success, particularly in pediatric populations.

Several explanations may account for this observed discrepancy. A key limitation is the lack of standardized MIC breakpoints for *E. meningoseptica*, which can result in poor concordance between laboratory findings and clinical outcomes. Additionally, antimicrobial resistance in this organism is largely mediated by β-lactamase expression, which may vary according to host factors and in vivo conditions, leading to differences between phenotypic resistance observed in vitro and actual antimicrobial activity in patients [3]. 

Pharmacokinetic and pharmacodynamic parameters may also contribute. In meningitis, inflammation-induced disruption of the blood-brain barrier and high-dose antimicrobial therapy may result in CSF antibiotic concentrations exceeding in vitro MIC values, potentially enhancing bactericidal activity despite apparent resistance [12]. Furthermore, in vitro studies have demonstrated additive or synergistic effects between β-lactams and fluoroquinolones against *Elizabethkingia* species, which may further augment antimicrobial efficacy in vivo [13]. For example, cefotaxime and levofloxacin have demonstrated synergy in experimental meningitis models, with the combination providing greater bactericidal activity and suppressing the selection of levofloxacin-resistant mutants [12].

Existing literature has also highlighted significant discordance between in vitro and in vivo antimicrobial responses in this pathogen, particularly to vancomycin. Di Pentima et al. reported three neonates with *E. meningoseptica* meningitis who responded successfully to vancomycin plus rifampicin, despite documented vancomycin resistance in vitro. This clinical success correlated with excellent in vitro synergy data for vancomycin plus rifampicin against four *E. meningoseptica* strains [13]. Additionally, in outbreak investigations by Ceyhan et al., three of four neonates survived with vancomycin plus oral rifampicin, with isolates showing in vitro resistance to vancomycin [14]. 

In most other literature, however, *E. meningoseptica* infection in neonates was treated with antibiotics selected based on susceptibility profiles. In one such report by Arbune et al. describing a case of *E. meningoseptica* meningitis in a preterm female infant, initial empiric therapy with gentamicin and ampicillin was ineffective. The regimen was escalated to meropenem and vancomycin, then adjusted to piperacillin/tazobactam and rifampicin based on susceptibility, with vancomycin retained. The patient required six weeks of antibiotics and neurosurgical drainage for hydrocephalus [15]. A retrospective case series by Goel et al. described seven neonates, most of whom were preterm, with meningitis due to *E. meningoseptica*. The isolates were resistant to piperacillin-tazobactam, aminoglycosides, meropenem, and colistin. Treatment was individualized, often involving prolonged courses of antibiotics to which the organism was susceptible in vitro, such as vancomycin and fluoroquinolones. Despite therapy, mortality was high and survivors frequently developed hydrocephalus [16]. These reports make it clear that even the use of antimicrobials to which the organism is susceptible does not guarantee favorable outcomes in vivo.

Meningitis due to *E. meningoseptica* is often fatal, with mortality rates as high as 57%, and many survivors experience severe post-infectious complications [6]. In a review of pediatric cases from 1944 to 2017, 30.4% of survivors developed hydrocephalus, while others suffered motor and sensory deficits, seizures, spasticity, and hearing loss [5]. Remarkably, our patient made a full recovery with no signs of neurological sequelae at discharge or follow-up, as confirmed by clinical evaluation and ultrasonography. Given the high morbidity and mortality associated with this infection, his excellent outcome suggests that the choice of antibiotics was likely an important factor. The initiation and continuation of cefotaxime and ampicillin at the maximum dose appropriate for meningitis, despite in vitro resistance, may have been a key factor in this success.

Future research should further explore the relationship between in vitro antimicrobial resistance and in vivo treatment outcomes in *E. meningoseptica* infections. Specifically, studies should investigate whether resistance patterns observed in laboratory testing reliably predict clinical failure or if certain antibiotics may still be effective in vivo despite laboratory resistance. Such findings could help refine treatment strategies and improve outcomes, particularly in vulnerable pediatric patients.

## Conclusions

This case underscores the significant mortality risk of *E. meningoseptica* in neonates but also highlights the potential for successful outcomes with individualized treatment strategies. Our patient’s full recovery without neurological sequelae is a remarkable outcome, reinforcing the importance of considering clinical response in antibiotic selection, even when in vitro resistance is reported. Future research should explore mismatches between laboratory resistance profiles and real-world clinical efficacy, particularly in pediatric populations.
